# Quality of life in adults with lymphedema cholestasis syndrome 1

**DOI:** 10.1186/s12955-018-0972-1

**Published:** 2018-07-25

**Authors:** Kristin Iversen, Lill Monica Drivdal, Kristin J. Billaud Feragen, Amy Østertun Geirdal

**Affiliations:** 1 Centre for Rare Disorders, Oslo University Hospital, Rikshospitalet, Postboks 4950 Nydalen, 0424, Oslo, Norway; 2 Department of Clinical Service, Oslo University Hospital, Ullevål, Postboks 4956 Nydalen, 0424, Oslo, Norway; 3 Faculty of Social Sciences, Oslo and Akershus University College of applied Sciences, Postboks 4 St. Olavs plass, 0130, Oslo, Norway

**Keywords:** Lymphedema cholestasis syndrome 1, LCS1, Aagenaes syndrome, Lymphedema, Health related quality of life, SF-36, Cantril’s ladder, The Kaasa test, Rarity

## Abstract

**Background:**

LCS1 (Lymphedema Cholestasis Syndrome 1/Aagenaes syndrome) is a rare, hereditary disorder, where the highest known prevalence is in Norway. The disorder is characterized by lymphedema and periodic cholestasis from birth or the neonatal period. This study aimed to examine internal reliability of the SF-36, in addition to the group’s overall- and health related quality of life (OQoL and HRQoL) and psychosocial well-being.

**Methods:**

Twenty adults (aged 18–65) in Norway have been diagnosed with LSC1. Eighteen of these patients were included in the study and completed four questionnaires on overall and health related quality of life and psychosocial well-being: Cantril’s Ladder (CL), The Kaasa Test, the SF-36, and a lymphedema anamnesis questionnaire. Demographic data were registered, and 15 of the patients underwent a physical examination of the lymphedema. SF-36 scores were compared with those of 360 age and gender matched controls drawn from an earlier survey of the Norwegian general population. The Mann-Whitney U test and Chi-square (χ^2^) test were used to test internal differences in the patient group.

**Results:**

Health-related quality of life (HRQOL) was significantly reduced in patients with LSC1 compared to controls, in three out of eight areas, role physical, general health and mental health. Females scored significantly better than males in the patient group in two areas of SF-36, in CL, and in one of three scales of The Kaasa Test. Severe lymphedema was found to be significantly correlated to bodily pain and reduced mental health. The level of education was positively correlated to mental health.

**Conclusion:**

Overall quality of life (OQoL), health related quality of life (HRQoL) and psycho-social well-being were good in the patient group, but some dimensions of HRQoL were reduced. More severe extent of lymphedema was associated with poorer HRQoL.

## Background

Lymphedema cholestasis syndrome 1 (LCS1), or Aagenaes syndrome, is a rare inborn, hereditary disease, first described by Aagenaes et al. in 1968 [1]. Until life-prolonging treatments were identified, LCS1 was considered deadly or considerably life-shortening [1, 2]. There are approximately 100 registered patients with LCS1 worldwide. Norway has the highest known prevalence in the world, as there are 48 cases identified from 1968 and up to date. Nearly all Norwegian patients come from the same small geographic area in the south west of the country. The occurrence of the cases is consistent with an autosomal recessive inheritance [3]; however, the exact mutation is not yet identified.

LCS1 is characterized by cholestasis, an arrest in the flow of bile, typically from birth or in the neonatal period. The first long-lasting cholestasis usually improves spontaneously in preschool or the first years of primary school and then becomes episodic. These episodes may last from two to 6 months, vary in severity, and have been observed from one to eight times over the life span [3].

Besides jaundice and itching, untreated cholestasis causes malabsorption of fat and fat-soluble vitamins, which can result in bleeding tendency and skeleton abnormalities. Liver function tends to improve and become close to normal over time, but in some cases cholestasis can cause cirrhosis and liver failure [3, 4]. In Norway six of the patients (12%) have undergone liver transplantation [1, 3]. Treatment of liver disease in LCS1 comprises medication, diet and vitamin substitution. When the liver function normalizes, the diet may return to the normal or close to normal [2, 3, 5].

All patients are either born with or develop in early childhood a prominent lymphedema, mainly in the lower limbs, but also in other parts of the body. Lymphedema occurs when the lymphatic system is not able to transport the interstitial fluid back to the circulatory system, resulting in the thickening of the skin and the subcutaneous tissues [6, 7]. If not treated, the lymphedema will increase, become extensive and debilitating, and may lead to chronic damage of the tissue. Treatment varies by severity, and may consist of decongestive lymphatic therapy^1^and/or use of intermittent pneumatic pump, which are both time consuming and bothersome for the patient. If the need for lymphedema treatment is less pronounced, individual adjusted compression garments can be used [2, 8].

Although the LCS1’s impact on patients’ bodily appearance and physical functioning may have consequences for quality of life (QoL), no studies of such consequences exist.

Nevertheless, increased treatment possibilities in chronic and rare diseases coupled with greater appreciation of QoL as an important outcome measure has led to growing interest in research on QoL in rare diseases. This reflects that the objective of treatment in chronic diseases is not merely to reduce symptoms, but also to support the patient in living with the disease. QoL measures provide insights into how health problems shape patients’ perceptions of their life situation [9, 10]. Better understanding of the quality of life implications of LCS1 would help improve health service provision and information on how to live with this very rare diagnosis, and may also be valuable to other patient groups who experience similar symptoms or conditions.

The present study had two aims. First, we wanted to examine internal reliability of the validated self-administered questionnaire of quality of life (SF-36 v. 2) in this patient population. Second, the study aimed to examine overall- and health related QoL and psychosocial well-being in persons with LCS1 and to compare the LCS1 group to a normative sample from the general population. The impact of background variables such as gender and educational level on the outcome variables was investigated. Further, the difference between objective and subjective assessments of lymphedema was explored.

## Methods

Measuring QoL in rare diseases is challenging because of the small number of patients introduces potential sample bias and large confidence intervals [10]. Moreover, comparing QoL in patients with different diseases is challenging because different diseases cause different problems. Generic QoL instruments, such as the widely used Short Form-36 (SF-36), enable comparisons across populations; and are the main reasons why we chose this instrument for our study. However, such generic instruments do not necessarily capture all the important problems associated with a specific disease, nor do they assess the importance of each problem [11]. Further the instruments may not discriminate well enough, which can cause floor or ceiling effects. Disease specific instruments overcome some of these problems by offering the possibility to assess inter-population differences, but this does not apply for many of the rare diseases. There is little consensus on concepts or standard tools in this area, which has resulted in utilization of a wide range of instruments [9–13]. Besides the lack of disease- specific instruments, one reason to employ several tools in the research, is the assumption that it will provide a more complete picture of the study population and strengthen the study.

### Participants

#### Patients

Among the 48 Norwegian cases identified up to date, 20 adult patients were alive in 2009. We identified these patients from the patient registry at the Centre for Rare Disorders at Oslo University Hospital,^2^ and from Dr. Øystein Aagenaes personal knowledge. All of them (11 male and 9 female, age range 18–65 years) were invited to participate in this study, where the researchers provided an information letter, an informed consent form and the self-administrated questionnaires, together with a stamped envelope for the return of the questionnaires. A total of 18 patients (90%) gave their written consent to participate in the study, 11 (61% men and 7 (39%) women. Two young women chose not to participate, one because of severe illness and one did not give any reason. Three of the 18 participants (two men and one woman) did not want to take part in the physical assessment of the lymphedema, and therefore only contributed to the QoL survey. Their age was in the middle to the higher range of the sample.

#### Normative sample

Statistics Norway administered the SF-36 to a national sample within the adult population in 2002. A sample of 360 individuals (20 controls per patient with LCS1) were randomly drawn from this national sample, matching age and gender of the patients (females: 39%, *n* = 140 and males: 61%, *n* = 220).^3^ The sample size was chosen to ensure variation in answers corresponding to the normal population and to decrease statistical uncertainty.

#### Ethics

The Regional ethics committee of Southern Norway approved the study. It was conducted in accordance with the Declaration of Helsinki.

### Measures

#### Demography

Age, level of education, marital status and work status (or receiving disability benefits) was registered through a questionnaire.

#### Disease-related variables

##### Lymphedema anamnesis and experience

The severity and impact of the lymphedema was graded in two ways; one based on the patient’s subjective experience, and the other one based on a clinical examination and evaluation. Subjective evaluation was measured through five questions from a self-administered questionnaire about extent, annoyance, feeling of pain and/or heaviness, extent of treatment and therapeutic and/or relieving actions. The answers were given in a two, three or five graded scale. The alternative answers were “Yes”, “No” or “Sometimes”, one item was answered in frequency per year, and one by “Daily”, “Weekly”, “Sometimes”, “Seldom” or “Never”. Dichotomous answers were graded 0 or 4, three-point scales were graded 0, 2 or 4 and five-point scales were graded 0,1,2,3 or 4, where the highest score indicates more severe subjective affliction. Scores were summarized (0–20) so that the participants could be grouped into the two categorical variables; “Severe subjective affliction” (11–20) or “Minor to moderate subjective affliction” (0–10). In addition, a trained physician performed a clinical examination and grading constructed on scores (range 0–20) based on an inspection and palpation of the skin, effect of limb elevation and function. This grading was added to scores from Colour Duplex Ultrasound, which incorporates two elements, structure and flow, and such make it possible to evaluate the extent of lymphedema. Scores from the ultrasound also ranged from 0 to 20 depending on how many body parts were affected by the lymphedema; legs, arms, trunk and face [14]. Based on the objectively measured and clinical evaluated extent of the lymphedema, the scores were dichotomised into the two categorical variables: “Extensive objective lymphedema” (21–40) or “Minor to moderate objective lymphedema” (0–20). The comparison of lymphedema burden with these variables is made for this patient group only.

##### Cantril’s self-anchoring ladder

*Overall quality of life* was measured using the self-administered questionnaire Cantril’s Ladder (CL) [15–18]. The questionnaire is based on one item; “How is your life?” with ratings on an interval scale from 0 to10, where a score of 10 indicates the best possible quality of life. Cantril’s ladder intends to measure an overall evaluation of life satisfaction at the current time. The instrument has shown a satisfactory validity and reliability across time [19].

##### The Kaasa test

Psychosocial experiences of well-being were measured through the 12-item self-administered questionnaire The Kaasa test. The test consists of two parts; the first part contains ten items, five positively and five negatively worded statements, in order to avoid systematic response bias. The items are scored on a five-point Likert scale from 1 (highest well-being) to 5 (lowest well-being), and a mean total score based on the ten items is calculated. The questionnaire also includes two single items; happiness and general satisfaction (range 1–7), both with 1 as the highest value, based on the participants’ subjective assessment of the two preceding weeks. The questionnaire has been reported to have an acceptable level of validity and reliability [20, 21]. The scale’s internal consistency was good with a Cronbach’s α reported of 0.80.

##### The short form 36 (SF-36) version 2

The self-administered questionnaire Short Form 36 Version 2.0 (SF-36 v. 2) was used to measure health related quality of life (HRQoL). SF-36 is a generic, self-administered health indicator questionnaire proven to be suitable in several research and clinical settings. It is widely used and recognized as having good reliability and validity [22, 23]. The questionnaire has been translated and validated for use in Norwegian [24].The instrument is useful for measuring HRQoL, comparing different groups (general and specific), comparing patients’ relative burden of disease, treatment outcomes, and it is sensitive to changes in individual patients’ health status. The scores are based on 36 items and categorised into eight dimensions: physical function (PF), role physical (RP), bodily pain (BP), general health (GH), vitality (VT), social functioning (SF), role emotional (RE), and mental health (MH). The responses are processed into scores from 0 (lowest) to 100 (highest). In addition, two summary scales are calculated; the physical component scale (PCS) that includes PF, RP, BP and GH, and the mental component scale (MCS), that includes VT, SF, RE and MH. Both summary scales are norm-based scored, with a mean of 50 and a SD of 10 based on T-transformation (by a linear T-score standardization) [23].

To check whether the frequency of patients with low versus high HRQOL scores was significantly different in the LCS1 group compared to the norm group, a cut-off score in PCS and MCS was set at < 40, which is one standard deviation below the mean, and indicates cases with considerable poor HRQoL scores. This is an alternative method for identifying differences between groups, irrespective of similar mean scores.

##### Statistical analysis

Statistical analyses were performed using SPSS 18.0 (IBM Corp). Descriptive statistics were calculated to characterize the sample. The non-parametric Mann-Whitney U Test was chosen to compare QoL score between the LCS1 group and the norm population, due to lack of normal distribution (Shapiro-Wilk test *p* < 0.05) on the dependent variables in the LCS1 group, as well as the small sample. A drawback of the non-parametric test is that it can be less sensitive for true differences, (Type 2 error). Also the small sample size increases risk for this type of error [25, 26].

The chi square test was used to examine the association between the two categorical variables (objectively and subjectively rated extent of lymphedema) within the LCS1 group, and to compare the proportion of cases with less than 1 SD below mean between the LCS1 group and controls for the PCS- and MCS-variables. Given a small sample size, 1 SD was chosen as a cut-off point for poor health on SF-36. Significance levels were set at *p* < 0.05 for all analyses, also due to a small sample size.

## Results

Demographics, disease related outcomes, overall QoL (CL) and psychosocial well-being (The Kaasa Test) was obtained from the LCS1 group only.

### Internal validity

The internal consistency of SF-36 was good, except on the Vitality dimension in the LCS1 group, with Cronbach’s α values ranging from 0.56 (vitality) to 0.95 (role physical) in the LSC1 group, compared to values ranging from 0.88 (vitality and physical functioning) to 0.98 (role physical and role emotional) in the norm group.

### Demographics

Demographics are shown in Table 1. Marital status was comparable to the general population. Ten individuals (56%) were married or cohabiting, compared to 60% in the same age group in the Norwegian general population in 2009 [27]. Eight out of 18 (44%) had more than 13 years of education (university college or university), higher than in the corresponding age group in Norway in 2010 (36%), though not statistically significant [28].

**Table 1:** Demographics

		n (%)
Gender	Male	11 (61)
	Female	7 (39)
Age	19–29	5 (28)
	30–44	8 (44)
	45–65	5 (28)
Civil status	Single	8 (44)
	Married/cohabiting	10 (56)
Education	< 13 years	10 (56)
	> 13 years	8 (44)
Occupation	Work/student	13 (72)
	Disability benefit	5 (28)

The control group was matched for age and gender. No other demographic data were available

Thirteen of the 18 participants (71.5%) were either working or studying. However two of those (11%) had a partial disability pension. Five participants (28.5%) had a full disability pension.

### Disease-related outcomes

Based on objective measurements, 11 of 15 participants (73%) were categorised within the minor/moderate range, while four participants were categorised within the severe/extensive range. The patients’ subjective perceptions of severity were evenly distributed (7 and 8 respectively). The distribution is also showed in Table 2. The difference between objective and subjective assessments of severity were statistically significant (χ^2^ = 6.52, *p* < .05).

**Table 2:** Diseaserelated outcome

	Minor/moderate	Severe/extensive	*p*
	(n)	(n)	
Objective assessment	11	4	< .05
Subjective assessment	7	8	ns

### Overall quality of life – Cantril’s Ladder

Cantril’s Ladder QoL scores were in the higher range. Women reported significantly higher scores than men.

### Psychosocial well-being - The Kaasa Test

The low average mean scores reported on The Kaasa Test indicate that feelings of well-.

being, happiness and satisfaction were within the higher range. This complies with the scores on Cantril’s Ladder.

### Health-related quality of life (SF-36)

Similar as in the two previous tests, also for SF-36 there was a variation in participants’ answers on the scales. The results were not characterized by outliers, and no participants were excluded from the study population or from the control group. Mean scores were calculated for the total sample (Table 4), across gender (Table 5), educational levels (Table 6) and lymphedema assessments (Table 7).

Differences were found between the LCS1 group and controls on some of the subscales of the SF-36 (Table 4). The LCS1 group reported significantly lower health-related quality of life on the following subscales: Role Physical (MWU = 2500.0, *p* < .05), General health (U = 2251.5, *p* < .05), and Mental health (MWU = 2111.5,). Using χ^2^ (chi-square) tests (PCS and MCS Cases), a statistically significant difference also was found between the LCS1 group and controls on the physical component scale (PCS), where 27.8% of the patients scored below the cut-off point, compared to 11.1% in the norm group (*p* < 0.05). This indicates a higher frequency of lower QoL scores among patients than controls. No other differences were statistically significant (Table 4).

Two subscales differed significantly between males and females, with females reporting higher levels of general health (U = 16.0, *p* < .05) and better mental health, indicated by higher mental component score (MCS) (U = 16.0*, p* < .05) (Table 5).

Higher education is associated with significantly higher score in mental health (MH), than is lower levels of education (Table 6). No other differences between the groups were statistically significant (Table 4).

The disease-related variable objectively measured extent of lymphedema, and was significantly associated with bodily pain and mental health in the LCS1 group (Table 7) Differences in other subscales were not statistically significant. There were no statistically significant outcomes on the corresponding analyses on the subjective assessment of the lymphedema.

## Discussion

The study’s main finding is that overall health-related quality of life (HRQoL) was almost as good in patients diagnosed with LCS1 as in the control group. However, some variations in QOL were found. The LCS1 group had significant lower scores in the role physical, general health and mental health dimensions of SF-36 and in the physical components scale (PCS) compared to the control group. In addition, females scored significantly higher than males on the general health subscale and the mental health component score, which is supported by the corresponding findings of gender difference in the Cantril’s Ladder and The Kaasa Test. Further, the objective extent of the lymphedema was significantly associated with higher levels of pain and poorer mental health.

### Overall quality of life

Mean scores on overall quality of life were in the higher range. Cantrill’s Ladder scores were higher than those reported among people with epilepsy in Norway (CL: 5.31–7.88 reliant on subgroup) [29], in patients with Hereditary Hemorrhagic Telangiectasia in Norway (CL = 7.0) [16] and in a study on wealth and happiness in the general population across the world (CL = 5.36) [18]. The Kaasa Test mean scores were also higher than reported in two other studies describing disability and quality of life in patients with muscular dystrophy and post-polio syndrome respectively [30, 31]. This indicates that the patients in the LCS1 group perceived themselves to have good overall QoL and psychosocial well-being. The finding is in line with the general impression from the SF-36 questionnaire where only three dimensions (GH, RP and MH) were significantly reduced compared to the norm population, which is unusual, but not unique.

### Health related QoL

The significantly lower scores in HRQoL, on the dimensions role physical, general health and mental health compared to the control group demonstrate that the LCS1 group has health problems that affect both physical and mental QoL. This is in line with former findings in groups of patients with other chronic diseases [11, 16, 29, 32]. Clinical experience indicates that patients’ most salient problems are related to limited physical function, discomfort, feeling of heaviness and/or pain, and a troublesome and time-consuming treatment that first and foremost seems to be connected to the objective extent of the lymphedema. Also, disabilities characterized by problems of movement often impact quality of life quite negatively [33]. In our study, this was reflected in an association between objective assessments of the lymphedema and corresponding physical and psychological aspects of the disease, such as pain and reduced mental health, and also a significantly lower physical component score on the PCS case test. Among people living with hereditary haemorrhagic telangiectasia, additional disease-related symptoms were associated with reduced overall QoL, HRQoL as well as disease specific QoL, where pain was the most significant variable associated with reduced QoL on all levels [16].

Despite having considerable health and QoL challenges, the LCS1 group had similar mean scores as the controls on the social functioning scale, which may be an important finding. There are discrepancies between different QoL studies, and a lack of connection between the degree of physical impairment and overall QoL [11]. It is widely accepted that social support, belonging and emotional contact function as buffers against life stressors [34]. Belonging to a rare group where social interaction is prominent, such as The Norwegian LCS1 patient interest group, may strengthen positive feelings such as meaning and belonging. Participation in work life or studies is also known to contribute to social contact and integration [35], and could also help to explain the higher QOL among people living with LCS1 in Norway.

### Comparisons between LCS1 and other diseases with similar health challenges

Comparisons of the quality of life impact of LCS1 and other diseases with similar health challenges can help to identify the distinctive features of LCS1 group that may contribute to a good QoL in many domains despite considerable symptoms and health-related difficulties. Several interesting quality of life studies have been carried out among patients with symptoms comparable to those of LCS1, e.g. Marfan syndrome [36, 37], lymphedema of the lower limbs [38, 39] and Rheumatoid Arthritis [40].

In a Norwegian study of HRQoL in patients with Marfan syndrome [36], scores on all dimensions were considerable poorer than for LCS1, most clearly in the physical subscales of SF-36. In addition to pain and fatigue, this was attributed to the burdens connected to an uncertain disease- and life course, physical restrictions and stigma.

Rheumatoid Arthritis (RA) is a disease with similar symptoms and problems as LCS1. Both a Dutch [40] and a Norwegian [41] study reported considerably poorer health HRQoL than we found among LCS1 patients. Although there is a higher burden of pain in RA, the difference in findings still seems difficult to explain. A notable dissimilarity between the two diagnoses, which may account for some of the difference in findings, is that LCS1 is present from birth and symptoms become stable or decrease in adult age, while RA, usually starts later in life and is associated with progressive disability. This may contribute to a stronger negative impact on quality of life.

Studies of patients with lymphedema in the lower limbs are interesting to appraise in the context of LCS1, since the lymphedema usually are the most burdensome symptoms after adolescence and puberty. Several studies show higher HRQoL scores (in many domains) after treatment, though other factors than treatment may have influenced this outcome [38, 39, 42].

### Rarity of condition and quality of life

The rarity of a condition could potentially have a considerable influence on patients’ quality of life. This may result from widespread lack of knowledge about rare conditions among health professionals, which leads them to avoid, consciously or not, familiarizing themselves with the patient’s diagnosis or problems, compromising necessary examinations or treatment. In addition, lack of knowledge leads to a lower quality or lack of necessary advice on how to live and cope with the problems following the disease. Patients’ repeated experiences of health workers’ inadequate knowledge of their condition can lead them to distrust health professionals, and to feelings of being unimportant, neglected and not worthy [43–47]. Other aspects are feelings of loneliness and lack of opportunities to share experiences with other people with the same disorder or problem [43, 47, 48], which may affect specifically but not exclusively psychosocial dimensions of quality of life. However, in some rare genetic disorders with substantial physical impairment, patients’ QoL is unexpectedly good, even better than that of unaffected peers [11], and having a rare diagnosis may have some positive effects, especially the positive implications of being a member of a support group [44].

Further, one can assume that coping strategies may be of central importance. Notably, acceptance; optimism and hopefulness are associated with higher QoL, and psychological adaptation and ability to cope with stressors (the symptoms and challenges of the disease) appear to be important [11]. We did not measure coping strategies and adaptation, but these could be underlying variables explaining the positive QoL outcomes among people living with LCS1, and requires further in-depth research. It may also be reasonable to assume that having a disorder from birth on enhances the possibilities of adaption, especially when symptoms are quite stable.

### Differences within the LCS1 group

Females were found to report significantly higher general health (GH) and mental component score (MCS) dimensions on the SF-36 than males, and were supported by the results from Cantril’s Ladder and the Happiness subscale on The Kaasa Test. Most studies report higher or similar scores in males compared to females [49–55]. In the case of LCS1, this may be because the disease affects male’s general health negatively, though this has not been reported previously. Another possible explanation could be that the females with LCS1 in the present sample had better compliance to the treatment. Females may possibly also care more about their appearance, which could become an extra motivation to comply with burdensome treatment procedures.

The positive correlation found between the level of education and mental health is common and is often explained by the effect of education on work participation, career possibilities, income and status [35, 56–59]. The LCS1-group’s participation in studies or work life was quite high (72%) considering the burden of symptoms and exertions connected to the diagnosis [16]. It may be that the patients were especially aware of the necessity of education to get a suitable job, as has been found among patients with Marfan syndrome [37].

### Strengths and limitations

This is, to our knowledge, the first study of quality of life among adults living with LCS1. Although the results are interesting, the study has several limitations. The small sample size raises challenges related to statistical testing and analysis, including wide confidence intervals that can mask existing differences between subgroups. On the other hand, the LCS1 group consisted of nearly all adults in Norway affected by this diagnosis, and results are therefore likely to be representative of this specific patient group. Nevertheless, one or two subjects could have had a disproportionate influence on the findings. As can be seen in Tables 3, 4, 5, 6 and 7, the standard deviation (SD) ranged from 0.0 to 50.0 in the LCS1 group, demonstrating the heterogeneity within the sample. Furthermore, the vitality dimension had a low Cronbach’s alpha, indicating less than ideal reliability, as is common when a scale consists of few items and/or the items do not measure the same underlying construct [25].

**Table 3:** Overall QoL and Psychosocial well-being

	Total (*n* = 18)	Females (*n* = 7)	Males (*n* = 11)	*p*
	Mean (SD)	Mean (SD)	Mean (SD)	
Cantril’s Ladder (CL)	8,0 (1,4)	8,8 (1,1)	7,5 (1,5)	< .05
The Kaasa Test				
Well-being	1,9 (0,9)	1,7 (0,3)	2,1 (0,5)	ns
Happiness	2,3 (1,1)	1,6 (0,5)	2,7 (1,1)	< .05
Satisfaction	2,1 (1,0)	1,6 (0,6)	2,5 (1,0)	ns

**Table 4:** Health-related quality of life the LCS1 group compared to norm

Health-related quality of life	Study sample (*n* = 18)	Control group (*n* = 360)		
	Mean (SD)	Mean (SD)	MWU	*p*
Physical functioning (PF)	89.2 (12.3)	91.6 (14.7)	2512.0	ns
Role physical (RP)	66.7 (44.6)	85.4 (30.1)	2500.0	< .05
Bodily pain (BP)	76.2 (25.2)	80.1 (23.1)	2953.5	ns
General health (GH)	68.5 (22.4)	79.3 (19.6)	2251.5	< .05
Vitality (VT)	61.9 (10.7)	62.0 (19.3)	3005.0	ns
Social functioning (SF)	91.0 (15.3)	88.5 (19.4)	3121.0	ns
Role emotional (RE)	81.5 (36.6)	87.5 (29.2)	3064.0	ns
Mental health (MH)	75.8 (8.2)	80.7 (14.7)	2111.5	< .05
PCS	48.9 (9.8)	52.2 (8.4)	2512.0	ns
PCS Case < 40 (%)	27.8	11.1		< .05
MCS	51.6 (3.9)	51.5 (8.6)	2687.0	ns
MCS Case < 40 (%)	0	10.3		ns

*MWU* Mann-Withney U

**Table 5:** Differences in health related quality of life across gender in the LCS1 group

SF 36	Females (*n* = 7)	Males (*n* = 11)		
	Mean (SD)	Mean (SD)	MWU	*p*
Physical functioning (PF)	86.4 (16.6)	90.9 (9.4)	35.0	ns
Role physical (RP)	82.1 (37.4)	56.8 (47.6)	27.0	ns
Bodily pain (BP)	81.6 (21.7)	72.9 (27.6)	32.5	ns
General health (GH)	82.0 (13.5)	59.9 (23.1)	16.0	< .05
Vitality (VT)	65.0 (5.0)	60.0 (13.0)	25.5	ns
Social functioning (SF)	92.9 (18.9)	89.8 (13.5)	29.0	ns
Role emotional (RE)	100.0 (0.0)	69.9 (43.3)	24.5	ns
Mental health (MH)	79.4 (4.3)	73.5 (9.3)	21.5	ns
Physical Component score (PCS)	50.8 (10.4)	47.6 (9.7)	38.0	ns
Mental Component Score (MCS)	54.0 (2.3)	50.0 (3.9)	16.0	< .05

*MWU* Mann-Withney U

**Table 6:** Differences in health related quality of life across levels of education

SF 36	Low (*n* = 10)	High (*n* = 8)		
	Mean (SD)	Mean (SD)	MWU	*p*
Physical functioning (PF)	90.0 (10.7)	86.9 (14.4)	31.5	ns
Role physical (RP)	62,5 (46.0)	71.9 (45.2)	35.0	ns
Bodily pain (BP)	76.4 (21.9)	76.1 (30.4)	38.0	ns
General health (GH)	66.9 (24.4)	70.5 (21.2)	38.5	ns
Vitality (VT)	58.5 (9.7)	66.3 (10.9)	24.5	ns
Social functioning (SF)	93.8 (12.1)	87.5 (18.9)	34.5	ns
Role emotional (RE)	76.7 (38.7)	87.5 (35.4)	34.0	ns
Mental health (MH)	72.0 (8.8)	80.5 (4.0)	15.5	< .05
Physical Component score (PCS)	49.3 (9.1)	48.3 (11.3)	33.0	ns
Mental Component Score (MCS)	50.0 (3.7)	53.6 (3.1)	18.0	ns

*MWU* Mann-Withney U

**Table 7:** Differences in health related quality of life across objectively measured extent of lymphedema

SF 36	Minor/moderate (*n* = 11)	Severe/extensive (*n* = 4)		
	Mean (SD)	Mean (SD)	MWU	*p*
Physical functioning (PF)	89.1 (13.4)	87.5 (13.2)	20.6	ns
Role physical (RP)	81.8 (31.8)	25.0 (50.0)	9.0	ns
Bodily pain (BP)	86.8 (18.8)	48.8 (24.2)	4.5	< .05
General health (GH)	72.3 (18.3)	52.3 (27.8)	11.5	ns
Vitality (VT)	63.2 (10.1)	56.3 (13.8)	16.5	ns
Social functioning (SF)	90.9 (15.9)	84.4 (18.8)	17.5	ns
Role emotional (RE)	93.9 (20.1)	58.3 (50.0)	12.5	ns
Mental health (MH)	77.1 (7.4)	68.0 (8.6)	7.0	< .05
Physical Component score (PCS)	51.4 (8.6)	40.7 (10.4)	12.0	ns
Mental Component Score (MCS)	52.4 (3.4)	49.1 (5.5)	14.0	ns

*MWU* Mann Withney U. Three participants did not want to take part in the physical part of the study, restricting the sample size to *n* = 15 for this outcome measure

One of the outcome measures, the Kaasa test, has been shown to have satisfactory psychometric properties [20]. However, the measure is not well-known, and may therefore result in a limited addition to research for this patient group.

Information about psychometric properties for Cantril’s ladder was unfortunately not found, and data from control groups were not available for this measure, or for The Kaasa Test. Therefore, conclusions regarding these two outcome measures should be drawn with caution.

A challenge when comparing our sample with similar groups was to find a sufficient number of relevant studies. Furthermore, differences in instruments and outcome measures, different age groups, and widely varying diseases and problem areas, complicated comparisons between studies.

## Conclusion

Our study provides new and interesting knowledge on quality of life for a group of people with the very rare diagnosis, LCS1. The most important finding was that people with this diagnosis nevertheless had high scores in overall QoL and psycho-social well-being. In three areas of HRQoL, they had significantly lower scores than the control group. In addition, severe lymphedema was found to have a negative impact on HRQoL, probably explained by its association with pain and discomfort. Encouraging compliance with treatment may therefore benefit QoL. Having a high level of education was also associated with higher QoL, perhaps because education is important for getting a suitable job, and in turn for social integration, income and status. The gender differences we identified warrant closer investigation.

## Footnotes

1 The treatment includes manual lymphatic drainage (special physiotherapy), decongestive movement exercises, multilayer compressive bandaging and meticulous skin care.

2 The centre is a division of Oslo University Hospital and a national undertaking on behalf of the Norwegian Directorate for Health.

3 The “Survey of level of Living 2002 – Cross-sectional Theme: Health”. Statistics Norway (SSB) was responsible for collecting the data. The survey was funded by SSB, the Norwegian Institute of Public Health and the Department of Public Health and General Practice. Data were processed and anonymized for use by the Norwegian Social Science Data Services (NSD). SSB, the Norwegian Institute of Public Health, the Department of Public Health and General Practice, and NSD are not responsible for the analyses or interpretation of data in this study.
